# Effect of supplementation of feed with Flaxseed (*Linumusitatisimum*) oil on libido and semen quality of Nilli-Ravi buffalo bulls

**DOI:** 10.1186/s40781-016-0107-3

**Published:** 2016-07-01

**Authors:** Syed Mazhar Hussain Shah, Shujait Ali, Muhammad Zubair, Huma Jamil, Nazir Ahmad

**Affiliations:** Department of Theriogenology, Faculty of Veterinary Sciences, University of Agriculture Faisalabad, Faisalabad, Pakistan

**Keywords:** Semen, Flax seed and breeding bulls

## Abstract

**Background:**

The current study was designed to investigate the effect of supplementation of Flaxseed (*Linumusitatisimum*) oil on libido and semen quality of Nilli-Ravi buffalo bulls.

**Methods:**

In this study, 12 adult healthy bulls kept at the Semen Production Unit, Qadirabad district Sahiwal, were used. These bulls were divided into three equal groups, A, B and C. Group A was kept as control, while in groups B and C supplementation of feed was provided by using flaxseed oil @125 ml/day and 250 ml/day,respectively for 12 weeks. Two ejaculates per animal were collected at 0 day then 5th, 6th, 7th, 8th, 9th, 10th, 11th and 12th week of treatment. In this way a total 216 samples were taken, and each semen sample was evaluated for color, volume, mass activity, percent motility, sperm cell concentration per ml, percentage of live sperm, and plasma membrane integrity. Libido of bulls was also evaluated before every collection.

**Results:**

Analysis of data revealed that these parameters were significantly (*P* < 0.01) increased in flax oil treated animals as compared to control.

**Conclusion:**

It was concluded from the present study that flax seed oil has beneficial effects on reproductive health of buffalo bull.

## Background

Male contributes 50 % of the herd and importance of male in genetic improvement of livestock cannot be denied. Genetic potential of a male is commonly exploited through artificial insemination. Reproductive capacity of males is judged by sperm production and success of artificial insemination depends upon number and quality of sperms in the ejaculate [6]. Semen is collected from genetically superior animals. In addition to other factors, volume of semen, sperm concentration, motility and number of normal sperm have great importance in conception.

In Mammalian sperm, lipids especially n-3 fatty acids are dominantly present [24]. These fatty acids are involved in motility of sperm, integrity of sperm membrane and protection of sperm from cold shock [25]. Animals are unable to synthesize the n-3 and n-6 fatty acids in their body as they do not have sufficient amount of fatty acid denaturize enzymes. Therefore, these fatty acids need to be taken by dietary sources [31]. Previous studies have shown that those fatty acids which are present in diet are also a part of sperm in human [8] and in farm animals [16]. Moreover, ration having fatty acids in diet improves value of post thawed buffalo bull semen [1].

Docosahexaenoic acid has been shown to increases serum testosterone level and sperm motility in man [8], boar [26], goat [9] and sheep [30] and flaxseed is a source of docosahexaenoic acid. Omega -3 fatty acids are necessary for cell membrane integrity and fitness of brain and eyes [8]. Main component of flaxseed is lignin, which contains plant estrogen and antioxidants and has protective effect on sex hormone metabolism [2].

Little information is available with regard to the effect of dietary flax seed oil supplementation and fertility of buffalo bulls during heat stress . The objective of present study was to investigate the effect of flaxseed oil supplementation on libido and semen quality parameters of Nilli-Ravi buffalo bulls.

## Methods

### Experimental animals

A total of 12 adult Nilli-Ravi buffalo bulls with clinically normal reproductive tract kept at the Semen Production Unit Qadirabad district Sahiwal, Pakistan were used in this study. These bulls were routinely used for semen collection. Animals were randomly divided into 3 equal groups A, B and C. Group A was kept as control while, group B and C was supplemented flaxseed oil drenched orally with dose of 125 ml and 250 ml respectively. All these treatments were continued for consecutive 12 weeks. The study period was from mid April to mid July, 2015. Semen was collected by artificial vagina at day 0 then 5th, 6th, 7th, 8th, 9th, 10th, 11th and 12th week of treatment. Two ejaculates per animal were collected on each collection day.

#### Parameters examined

Libido of each bull was assessed by mating behavior of bulls. Mating behavior was videotaped using video camera and video recorder. By watching videotapes libido in terms of reaction time to first semen collection was recorded [17]. Each ejaculate was evaluated for color, volume, mass activity, percent motility, sperm concentration, live sperm, and plasma membrane integrity. To examine mass activity, a small drop of semen was placed on pre warmed glass slide with glass rod and examined under microscope. On the base of wave pattern, mass activity score from0 to 5 was assigned to each sample [27]. To examine motility of spermatozoa, a drop of semen was placed on glass slide and mixed with a drop of 2.9 % sodium citrate. Slide was examined under 200X magnification. Sperm concentration was directly determined using Accucell Bovine Photometer. Sperm membrane functional integrity was determined by hypo-osmotic swelling test (HOST) which is based on semi permeability of cell membrane. The hypo-osmotic solution (150 mosm/L) was prepared as described by [19]. Eosin-nigrosin staining was used to determine live perm percentage.

#### Statistical analysis

Mean values (± SE) of various parameters for bulls of three groups were calculated. The differences in group were compared by “Tukey test”. The data was analyzed using SPSS software.

## Results

Analysis of variance showed that there is (*P* < 0.01) improvement of libido in treated groups as compared to control. Interaction between weeks and groups was also significant (Table 1). Analysis of variance of Table 2 revealed that there was the significant effect of weeks and treatment on semen volume (*p*˂0.01). Further analysis showed that semen volume for bulls of group B and C was (*p*˂0.01) higher than the control. However, difference between group B and C was non-significant. The effect of weeks and treatment on semen mass activity of treated groups was significant (*p*˂0.01) higher as in Table 3. Likewise, weekly analysis of variance revealed that semen mass activity for bulls of group B and C was significantly higher than the control. However, there was non-significant difference between group B and C.

**Table 1:** Mean values (±SE) of Libido collected from bulls of control and treatment groups on different weeks

Weeks of Treatments	Group			Over all Mean
	A (Control)	B (Treatment 1)	C (Treatment 2)	
0	7.50 ± 0.50a	7.25 ± 0.48ab	7.50 ± 0.29a	7.42 ± 0.23A
5	7.50 ± 0.65a	4.75 ± 0.25cde	4.25 ± 0.48e	5.50 ± 0.50B
6	6.75 ± 0.48a-d	5.00 ± 0.41b-e	4.75 ± 0.48cde	5.50 ± 0.36B
7	7.25 ± 0.48ab	4.50 ± 0.29de	4.25 ± 0.25e	5.33 ± 0.45B
8	7.50 ± 0.29a	4.50 ± 0.29de	4.25 ± 0.48e	5.42 ± 0.48B
9	7.00 ± 0.71abc	4.00 ± 0.41e	4.00 ± 0.41e	5.00 ± 0.51B
10	7.25 ± 0.48ab	4.25 ± 0.48e	4.00 ± 0.41e	5.17 ± 0.51B
11	6.75 ± 0.25a-d	4.00 ± 0.41e	4.00 ± 0.41e	4.92 ± 0.43B
12	7.00 ± 0.41abc	4.00 ± 0.41e	4.00 ± 0.41e	5.00 ± 0.48B
Mean	7.17 ± 0.15A	4.69 ± 0.20B	4.56 ± 0.22B	

Means sharing similar letters in a row or in a column are statistically non-significant (*P* > 0.05). Small letters represent comparison among interaction means and capital letters are used for overall means

**Table 2:** Mean values (±SE) of semen volume (ml) collected from bulls of control and treatment groups on different weeks

Weeks of Treatment	Group			Overall Mean
	A (Control)	B (Treatment 1)	C (Treatment 2)	
0	5.25 ± 0.95d-h	4.50 ± 0.29fgh	3.75 ± 0.48 h	4.50 ± 0.38D
5	3.38 ± 0.24 h	5.00 ± 0.41d-h	4.50 ± 0.29fgh	4.29 ± 0.26D
6	3.75 ± 0.48 h	5.75 ± 0.14c-g	4.75 ± 0.25e-h	4.75 ± 0.30CD
7	4.00 ± 0.41gh	6.50 ± 0.20a-e	6.00 ± 0.41b-f	5.50 ± 0.37BC
8	3.63 ± 0.38 h	6.50 ± 0.20a-e	6.75 ± 0.32a-d	5.63 ± 0.46ABC
9	3.63 ± 0.38 h	7.38 ± 0.24abc	7.38 ± 0.24abc	6.13 ± 0.55AB
10	3.75 ± 0.48 h	7.63 ± 0.24abc	7.88 ± 0.13ab	6.42 ± 0.59AB
11	3.63 ± 0.38 h	7.88 ± 0.31ab	8.00 ± 0.20a	6.50 ± 0.63A
12	3.63 ± 0.24 h	7.88 ± 0.13ab	8.13 ± 0.13a	6.54 ± 0.63A
Mean	3.85 ± 0.17B	6.56 ± 0.21A	6.35 ± 0.28A	

Means sharing similar letters in a row or in a column are statistically non-significant (*P* > 0.05). Small letters represent comparison among interaction means and capital letters are used for overall means

**Table 3:** Mean values (±SE) of Mass Activity collected from bulls of control and treatment groups on different weeks

Weeks of Treatment	Group			Overall Mean
	A (Control)	B (Treatment 1)	B (Treatment 2)	
0	1.75 ± 0.25ef	1.50 ± 0.29f	1.50 ± 0.29f	1.58 ± 0.15E
5	1.75 ± 0.25ef	2.25 ± 0.25def	2.00 ± 0.00def	2.00 ± 0.12DE
6	2.00 ± 0.00def	2.25 ± 0.25def	2.00 ± 0.00def	2.08 ± 0.08CDE
7	2.00 ± 0.00def	2.50 ± 0.29c-f	2.50 ± 0.29c-f	2.33 ± 0.14CD
8	1.75 ± 0.25ef	2.50 ± 0.29c-f	2.75 ± 0.25b-e	2.33 ± 0.19CD
9	1.75 ± 0.25ef	3.00 ± 0.00a-d	3.00 ± 0.00a-d	2.58 ± 0.19BC
10	2.00 ± 0.00def	3.50 ± 0.29abc	3.50 ± 0.29abc	3.00 ± 0.25AB
11	2.00 ± 0.00def	3.75 ± 0.25ab	4.00 ± 0.00a	3.25 ± 0.28A
12	2.00 ± 0.00def	4.00 ± 0.00a	4.00 ± 0.00a	3.33 ± 0.28A
Mean	1.89 ± 0.05B	2.81 ± 0.15A	2.81 ± 0.15A	

Means sharing similar letters in a row or in a column are statistically non-significant (*P* > 0.05). Small letters represent comparison among interaction means and capital letters are used for overall means

Analysis of variance indicated that motility percentage was highly significant (*P*˂0.01) in treated groups as compared to control. However, difference between group B and C was non-significant. Interaction between weeks and groups was also significant (Table 4). Analysis of variance reveled that sperm concentration is highly significant (*P* < 0.01) in both treated group as compared to control group (Table 5). However, difference between group B and C was non-significant. Interaction between weeks and groups was also significant. Mean value of live sperm percentage in control group A was lower as compared to b and C. Analysis of variance showed that there was significant effects on treated group as compared to control (Table 6). Overall mean value for HOST in control group A was lower as compared to treated groups (Table 7). Weekly analysis of variance showed that there was highly significant (*P* < 0.01) difference between treated and control group. Interaction between weeks and groups was also significant.

**Table 4:** Mean values (±SE) of Motility percentage collected from bulls of control and treatment groups on different weeks

Weeks of Treatments	Group			Overall Mean
	A (Control)	B (Treatment 1)	C (Treatment 2)	
0	68.75 ± 1.25c-f	65.00 ± 2.04ef	65.00 ± 0.00ef	66.25 ± 0.90C
5	63.75 ± 1.25f	70.00 ± 2.04b-f	66.25 ± 2.39def	66.67 ± 1.28C
6	67.50 ± 1.44def	68.75 ± 1.25c-f	67.50 ± 1.44def	67.92 ± 0.74BC
7	67.50 ± 1.44def	70.00 ± 2.04b-f	68.75 ± 1.25c-f	68.75 ± 0.90BC
8	66.25 ± 2.39def	73.75 ± 1.25a-e	75.00 ± 2.04a-d	71.67 ± 1.55AB
9	66.25 ± 2.39def	77.50 ± 1.44abc	77.50 ± 1.44abc	73.75 ± 1.86A
10	66.25 ± 2.39def	77.50 ± 1.44abc	77.50 ± 1.44abc	73.75 ± 1.86A
11	63.75 ± 2.39f	78.75 ± 1.25ab	80.00 ± 2.04a	74.17 ± 2.45A
12	61.25 ± 1.25f	81.25 ± 1.25a	82.50 ± 1.44a	75.00 ± 3.02A
Mean	65.69 ± 0.67B	73.61 ± 0.99A	73.33 ± 1.14A	

Means sharing similar letters in a row or in a column are statistically non-significant (*P* > 0.05). Small letters represent comparison among interaction means and capital letters are used for overall means

**Table 5:** Mean values (±SE) of Sperm Concentration (Million/ml) of semen collected from bulls of control and treatment groups on different weeks

Weeks of Treatments	Group			Overall Mean
	A (Control)	B (Treatment 1)	C (Treatment 2)	
0	1626.8 ± 38.0d-g	1001.9 ± 182.9 k	1246.0 ± 108.5 h-k	1291.5 ± 101.3 F
5	1266.0 ± 93.8 g-k	1429.8 ± 29.1f-j	1354.0 ± 24.9f-k	1349.9 ± 036.6EF
6	1094.8 ± 102.2jk	1591.5 ± 16.0e-h	1472.5 ± 13.8e-i	1386.3 ± 071.2EF
7	1176.0 ± 41.6ijk	1696.8 ± 26.1def	1644.0 ± 39.4def	1505.6 ± 073.1DE
8	1171.3 ± 92.8ijk	1844.5 ± 83.8cde	1811.0 ± 38.3cde	1608.9 ± 101.4CD
9	1171.3 ± 92.8ijk	2071.3 ± 56.2abc	1988.5 ± 39.2bcd	1743.7 ± 127.3BC
10	1270.0 ± 72.5 g-k	2356.0 ± 47.7ab	2105.8 ± 45.8abc	1910.6 ± 143.1AB
11	1243.3 ± 52.8 h-k	2371.8 ± 46.2a	2299.0 ± 59.4ab	1971.3 ± 157.9A
12	1196.3 ± 46.5ijk	2405.0 ± 47.4a	2378.0 ± 58.4a	1993.1 ± 172.0A
Mean	1246.2 ± 32.7B	1863.2 ± 80.2A	1811.0 ± 67.3A	

Means sharing similar letter in a row or in a column are statistically non-significant (*P* > 0.05). Small letters represent comparison among interaction means and capital letters are used for overall mean

**Table 6:** Mean values (±SE) of live percentage of spermatozoa collected from bulls of control and treatment groups on different weeks

Week of Treatments	Group			Overall Mean
	A (Control)	B (Treatment 1)	C (Treatment 2)	
0	83.75 ± 1.25a-d	77.50 ± 1.44 cd	76.25 ± 1.25 cd	79.17 ± 1.20BC
5	73.75 ± 2.39d	77.50 ± 1.44 cd	75.00 ± 2.04 cd	75.42 ± 1.14C
6	76.25 ± 2.39 cd	77.50 ± 1.44 cd	75.00 ± 2.04 cd	76.25 ± 1.09BC
7	73.75 ± 2.39d	77.50 ± 1.44 cd	76.25 ± 2.39 cd	75.83 ± 1.20C
8	76.25 ± 2.39 cd	80.00 ± 2.04bcd	82.50 ± 2.50a-d	79.58 ± 1.44BC
9	76.25 ± 2.39 cd	83.75 ± 2.39a-d	85.00 ± 2.89a-d	81.67 ± 1.78AB
10	73.75 ± 2.39d	86.25 ± 2.39abc	85.00 ± 2.89a-d	81.67 ± 2.16AB
11	73.75 ± 2.39d	90.00 ± 2.04ab	92.50 ± 1.44a	85.42 ± 2.71A
12	73.75 ± 2.39d	93.75 ± 1.25a	93.75 ± 1.25a	87.08 ± 2.98A
Mean	75.69 ± 0.85B	82.64 ± 1.12A	82.36 ± 1.33A	

Means sharing similar letters in a row or in a column are statistically non-significant (*P* > 0.05). Small letters represent comparison among interaction means and capital letters are used for overall mean

**Table 7:** Mean values (±SE) of sperm membrane functional integrity (%) of control and treatment groups on different weeks

Week of Treatments	Group			Overall Mean
	A (Control)	B (Treatment 1)	C (Treatment 2)	
0	52.50 ± 1.44hi	51.25 ± 1.25i	53.75 ± 2.39ghi	52.50 ± 0.97 F
5	51.25 ± 1.25i	55.00 ± 2.04ghi	53.75 ± 2.39ghi	53.33 ± 1.12 F
6	51.25 ± 1.25i	62.50 ± 1.44e-h	57.50 ± 3.23f-i	57.08 ± 1.79EF
7	52.50 ± 1.44hi	66.25 ± 1.25c-f	63.75 ± 2.39d-g	60.83 ± 2.03DE
8	52.50 ± 3.23hi	68.75 ± 1.25a-e	67.50 ± 1.44b-f	62.92 ± 2.50CD
9	52.50 ± 3.23hi	72.50 ± 1.44a-e	71.25 ± 2.39a-e	65.42 ± 3.04BCD
10	55.00 ± 4.08ghi	73.75 ± 1.25a-d	71.25 ± 1.25a-e	66.67 ± 2.84ABC
11	55.00 ± 2.04ghi	77.50 ± 1.44ab	76.25 ± 1.25abc	69.58 ± 3.23AB
12	55.00 ± 2.04ghi	78.75 ± 1.25a	78.75 ± 1.25a	70.83 ± 3.47A
Mean	53.06 ± 0.75B	67.36 ± 1.59A	65.97 ± 1.62A	

Means sharing similar letters in a row or in a column are statistically non-significant (*P* > 0.05). Small letters represent comparison among interaction means and capital letters are used for overall means

## Discussion

This study was planned to examine the effect of supplementation of feed with flaxseed oil on libido and quality parameters of Nilli- Ravi buffalo bull’s semen. Poly unsaturated fatty acids are mainly present in mammalian sperm which are not synthesized in body so they should be supplemented [31].

In the present study, libido in treated bulls was increased from 9 to 12 weeks of treatment. These values are in line with Estienne et al., [11] reported in boars. This increment in the libido may be due to production of higher amount of testosterone hormone. As testosterone is a steroid hormone and synthesized by cholesterol. Flaxseed oil having polyunsaturated fatty acids such as olic acids and linolinic acids which are major source of cholesterol [22]. In ram testosterone level increases in short day, but by supplementation of flaxseed oil same concentration is obtained in summer months (Baiomy and Mottelib, 20e409).

The volume of semen volume was increased in from 9 to 12 months of treatment as compared to control animals. Similarly, increase in semen volume was observed in goats [9], rams [5] and quails [4], Rooke et al., [26]. In contrary to present study Gliozzi et al., [14], Adeel et al., [1], Gholami et al., [13] reported non-significant increase in semen volume in chicken, rabbit, buffalo bulls and Holstein bulls respectively. Flaxseed contains high value of omega-3 and omega-6 fatty acids which acts as antioxidant.

In present study overall mean values of mass activity were higher due to flax seed. Jafaroghli et al., [18] reported that supplementation with omega-3 fatty acids lead to significantly increase in mass activity of human semen. Improvement in mass activity of semen may be due to higher concentration and viablity sperm.

The importance of sperm motility has achieved a central role in the routine diagnosis of male fertility [3]. Motility percentage was significantly higher (*p*˂0.01) in treatment groups as compared to control group during 8–12 weeks of trial. Similar kind of results are reported in human [8], pigs [26], rams [5], goat [9, 28], rats [32] and boars [20]. Contrary to this study Adeel et al., [1] reported no improvement in motility of semen of buffalo bulls. Increased in motility percentage of semen may be due to fatty acids composition of sperm tail as reported by Mourvaki et al., [21] that flaxseed seed supplementation increased poly unsaturated fatty acids in rabbit sperm. This study showed that tail is major portion which is affected by supplementation of flaxseed supplementation. It can be assumed that poly unsaturated fatty acids present in flaxseed oil are involved in flagellar movement of sperm which in turn increases the motility percentage. The overall mean values of sperm concentration of treated groups were higher as compared to control. These results were similar in Stallion [29], goat [9], boars [11, 12], rams (Baiomy and Mottelib [5]) and men [12]. This increment in the sperm concentration in present study may be due to rapid spermatogenesis and antiapoptosis properties of supplementation of omega -3 fatty acids in feed. Supplementation of poly unsaturated fatty acids has positive effect on biosynthesis of prostaglandin and steroidogenesis. Poly unsaturated fatty acids have ability to affect the hypothalamic—pituitary axis and hormonal control of spermatogenesis.

The viability of sperm is considered as the useful parameters to differentiate between fertility and infertility conditions of males [15]. In the present study, lives sperm percentage was increased from 8 to 12 weeks of treatment. These findings are in close harmony as described by Rooke et al., [26], Dolatpanah et al., [9], Baiomy and Mottelib, [5], Mourvaki et al., [21],Gholami et al., [13], Al-Daraji et al., [4] and Esmaeili et al., [10]. The more number of live spermatozoa in ejaculates may be due to inhibition of apoptosis by action of poly unsaturated fatty acids as seen in culture off retinal photoreceptor and neuron [23].

The intact functional membrane of sperm is considered as the prerequisite for the sperm fertilizing ability due to successful acrosome reaction and binding to egg surface [7]. The functional integrity of sperm increased from 9 to 12 weeks of treatment in treated animals as compared to control. Similar to this study, comparable results were present in rabbit buck [21], buffalo bull [1], and in Holstein bulls Gholami et al., [13]. Increase in HOST positive spermatozoa was may be due to effect of poly unsaturated fatty acid on integrity of membrane [1].

## Conclusion

It could be concluded that Supplementation of flaxseed oil has beneficial effect on semen quality parameter and libido of Nilli-Ravi buffalo bulls. This study further need to evaluate the effects of flax seed on cryopreservation and bull fertility.
